# Dietary sodium induces a redistribution of the tubular metabolic workload

**DOI:** 10.1113/JP274927

**Published:** 2017-10-15

**Authors:** Khalil Udwan, Ahmed Abed, Isabelle Roth, Eva Dizin, Marc Maillard, Carla Bettoni, Johannes Loffing, Carsten A. Wagner, Aurélie Edwards, Eric Feraille

**Affiliations:** ^1^ Department of Cellular Physiology and Metabolism University of Geneva CMU, 1 Rue Michel‐Servet CH‐1211 Geneva 4 Switzerland; ^2^ Centre hospitalier universitaire Vaudois Service de néphrologie CH‐1011 Lausanne Switzerland; ^3^ Institute of Physiology University of Zürich Winterthurerstrasse 190 CH‐8057 Zürich Switzerland; ^4^ Institute of Anatomy University of Zürich Winterthurerstrasse 190 CH‐8057 Zürich Switzerland; ^5^ Centre de Recherche des Cordeliers INSERM UMRS1138 and CNRS ERL8228 15 rue de l'Ecole de Médecine F‐75006 Paris France; ^6^ Department of Biomedical Engineering Boston University Boston MA USA; ^7^ National Centre of Competence in Research NCCRKidney CH Switzerland

**Keywords:** dietary salt, Na^+^ transport, Na^+^ transporters, diuretics, optimal Na^+^ diet

## Abstract

**Key points:**

- Body Na^+^ content is tightly controlled by regulated urinary Na^+^ excretion.
- The intrarenal mechanisms mediating adaptation to variations in dietary Na^+^ intake are incompletely characterized.
- We confirmed and expanded observations in mice that variations in dietary Na^+^ intake do not alter the glomerular filtration rate but alter the total and cell‐surface expression of major Na^+^ transporters all along the kidney tubule.
- Low dietary Na^+^ intake increased Na^+^ reabsorption in the proximal tubule and decreased it in more distal kidney tubule segments.
- High dietary Na^+^ intake decreased Na^+^ reabsorption in the proximal tubule and increased it in distal segments with lower energetic efficiency.
- The abundance of apical transporters and Na^+^ delivery are the main determinants of Na^+^ reabsorption along the kidney tubule.
- Tubular O_2_ consumption and the efficiency of sodium reabsorption are dependent on sodium diet.

**Abstract:**

Na^+^ excretion by the kidney varies according to dietary Na^+^ intake. We undertook a systematic study of the effects of dietary salt intake on glomerular filtration rate (GFR) and tubular Na^+^ reabsorption. We examined the renal adaptive response in mice subjected to 7 days of a low sodium diet (LSD) containing 0.01% Na^+^, a normal sodium diet (NSD) containing 0.18% Na^+^ and a moderately high sodium diet (HSD) containing 1.25% Na^+^. As expected, LSD did not alter measured GFR and increased the abundance of total and cell‐surface NHE3, NKCC2, NCC, α‐ENaC and cleaved γ‐ENaC compared to NSD. Mathematical modelling predicted that tubular Na^+^ reabsorption increased in the proximal tubule but decreased in the distal nephron because of diminished Na^+^ delivery. This prediction was confirmed by the natriuretic response to diuretics targeting the thick ascending limb, the distal convoluted tubule or the collecting system. On the other hand, HSD did not alter measured GFR but decreased the abundance of the aforementioned transporters compared to NSD. Mathematical modelling predicted that tubular Na^+^ reabsorption decreased in the proximal tubule but increased in distal segments with lower transport efficiency with respect to O_2_ consumption. This prediction was confirmed by the natriuretic response to diuretics. The activity of the metabolic sensor adenosine monophosphate‐activated protein kinase (AMPK) was related to the changes in tubular Na^+^ reabsorption. Our data show that fractional Na^+^ reabsorption is distributed differently according to dietary Na^+^ intake and induces changes in tubular O_2_ consumption and sodium transport efficiency.

## Key points

- Body Na^+^ content is tightly controlled by regulated urinary Na^+^ excretion.
- The intrarenal mechanisms mediating adaptation to variations in dietary Na^+^ intake are incompletely characterized.
- We confirmed and expanded observations in mice that variations in dietary Na^+^ intake do not alter the glomerular filtration rate but alter the total and cell‐surface expression of major Na^+^ transporters all along the kidney tubule.
- Low dietary Na^+^ intake increased Na^+^ reabsorption in the proximal tubule and decreased it in more distal kidney tubule segments.
- High dietary Na^+^ intake decreased Na^+^ reabsorption in the proximal tubule and increased it in distal segments with lower energetic efficiency.
- The abundance of apical transporters and Na^+^ delivery are the main determinants of Na^+^ reabsorption along the kidney tubule.
- Tubular O_2_ consumption and the efficiency of sodium reabsorption are dependent on sodium diet.

**Abbreviations**
ACC: acetyl‐CoA carboxylase
AMPK: adenosine monophosphate‐activated protein kinase
BP: blood pressure
CCD: cortical collecting duct
CD: collecting duct
CKD: chronic kidney disease
CNT: connecting tubule
cTAL: cortical thick ascending limb of Henle
DCT: distal convoluted tubule
ENaC: epithelial sodium channel
GFR: glomerular filtration rate
HSD: high sodium diet
IMCD: inner medullary collecting duct
LSD: low sodium diet
mTAL: medullary thick ascending limb of Henle
NSD: normal sodium diet
OMCD: outer medullary collecting duct
pACC: phosphorylated acetyl‐CoA carboxylase
PCT: proximal convoluted tubule
PST: proximal straight tubule
PT: proximal tubule
TAL: thick ascending limb of Henle
TNa: Na^+^ transport

## Introduction

Average daily Na^+^ intake in modern societies is largely higher than 2 g (87 mmol/24 h), the value recommended for adults by the World Health Organization 2012 guidelines (http://www.who.int/nutrition/publications/guidelines/sodium_intake/en/). A high salt intake is associated with adverse health outcomes in relation to increased blood pressure (BP) (Meneton *et al*. 2005). Furthermore, salt may play an essential role in the progression of chronic kidney disease (CKD), which is characterized by decreased glomerular filtration rate, albuminuria as well as glomerular and tubular‐interstitial fibrosis, both in a BP‐dependent and in a BP‐independent manner (Boero *et al*. 2002; Kotchen *et al*. 2013). Conversely, dietary salt restriction has been shown to reduce BP, albuminuria and kidney fibrosis (Lambers Heerspink *et al*. 2012). In contrast, the few randomized controlled trials available did not clearly demonstrate the benefits of salt restriction in the general population (O'Donnell *et al*. 2014). Moreover, recent studies suggested that both very low and high Na^+^ intakes are associated with increased mortality, consistent with a U‐ or J‐shaped association between urinary Na^+^ excretion and cardiovascular outcomes (Graudal *et al*. 2014; O'Donnell *et al*. 2014; Pfister *et al*. 2014). While not universally accepted (Cogswell *et al*. 2016), this non‐linear relationship between Na^+^ intake and cardiovascular outcomes may indicate that the human cardio‐renal system is best adapted to an optimal range of Na^+^ intake.

Arterial BP depends on intravascular volume, which is directly proportional to plasma Na^+^ content (Meneton *et al*. 2005). It has traditionally been accepted that under physiological conditions, plasma and interstitial compartments are in equilibrium and that any change in extracellular Na^+^ content elicits a proportional change in extracellular water volume so as to maintain body fluid osmolality constant. To keep plasma volume within narrow limits, urinary Na^+^ excretion must match as closely as possible dietary Na^+^ intake minus skin and digestive Na^+^ losses. This precise adaptation requires a continuous coordination between the filtered amount of Na^+^ and its tubular reabsorption (Greger, 2000). In healthy individuals, 1–3% of the filtered Na^+^ is not reabsorbed and is excreted in urine (Meneton *et al*. 2005). Na^+^ reabsorption takes place along the nephron and collecting duct, via transcellular and paracellular pathways (Féraille & Doucet, 2001). Transcellular Na^+^ reabsorption is a two‐step process: Na^+^ enters tubular cells via a segment‐specific apical transporter and is extruded by the basolateral Na,K‐ATPase (Féraille & Doucet, 2001). The first step is mostly mediated by the apical Na^+^/H^+^ exchanger NHE3 in the proximal tubule (PT) (Tse *et al*. 1992), the Na^+^/K^+^/2Cl^–^ cotransporter NKCC2 in the thick ascending limb of Henle's loop (TAL) (Gamba *et al*. 1994) and the NaCl cotransporter NCC in the distal convoluted tubule (DCT) (Gamba *et al*. 1993). Lastly, the fine‐tuning of Na^+^ reabsorption occurs via the multimeric Na^+^ channel ENaC under the control of aldosterone in the late DCT, connecting tubule (CNT) and collecting duct (CD) (Canessa *et al*. 1994).

In the kidney, ATP and oxygen consumption are determined primarily by the energy required for tubular Na^+^ reabsorption (Kiil *et al*. 1961; Brodwall & Laake, 1964; Mandel, 1986). Transcellular Na^+^ transport is directly dependent on the electrochemical potential generated by Na,K‐ATPase, which uses 1 molecule of ATP to drive the outward transport of 3 Na^+^ and the inward transport of 2 K^+^. AMP‐activated protein kinase (AMPK) is a key controller of cellular energy metabolism. AMPK activity, which is stimulated when the AMP/ATP ratio increases, reflects the intensity of active Na^+^ handling and links it to the metabolic status of a cell (Hallows *et al*. 2000).

The aims of the present study were to assess the respective role of changes in the abundance of the renal Na^+^ transporters and sodium delivery in the rate of Na^+^ transport by the different segments of the kidney tubule in response to different Na^+^ intakes, as well as the relationship between tubular Na^+^ transport and the activity of the metabolic sensor AMPK. We show in normal mice that in response to a low sodium diet (LSD), the expression levels of most apical Na^+^ transporters and Na,K‐ATPase were increased while Na^+^ transport decreased in segments downstream from the medullary TAL (mTAL). Under a high sodium diet (HSD), the expression levels of most apical Na^+^ transporters were decreased, and Na^+^ transport decreased in PT and most kidney tubules segments but increased in cortical TAL (cTAL) and CD in response to increased Na^+^ delivery. The rate of Na^+^ reabsorption by tubular segments was correlated with AMPK activity. Finally, the efficiency of tubular Na^+^ reabsorption with respect to O_2_ consumption was predicted to be slightly higher under normal sodium diet (NSD) as compared to LSD or HSD.

## Methods

### Animals

Male C57B6 mice (Charles‐River, Saint Germain de l'Arbresle, France) aged 12–16 weeks were fed for 7 days with either a low (0.01% w/w), standard (0.18% w/w) or moderately high sodium (1.25% w/w) diet (Provimi‐Kliba, Kaiseraugst, Switzerland). All diets contained the same amounts of potassium (0.8% w/w). To record physiological parameters, mice were placed in metabolic cages (Tecniplast, Buguggiate VA, Italy) under controlled conditions of light, temperature and humidity. After 2 days of adaptation, body weight, food intake, water intake and 24‐h‐period urine samples were measured and collected from day 0 to day 6 after the start of each sodium diet. Urinary creatinine, Na^+^, Cl^−^ and K^+^ concentrations were determined using ion‐specific electrodes (UniCel DxC800 Synchron Clinical System, Beckman Coulter, High Wycombe, UK). Absolute Na^+^, Cl^−^ and K^+^ excretions were measured. Urinary aldosterone levels were measured according to standard procedures using the Coat‐A‐Count RIA kit (Siemens Medical Solutions Diagnostics, Ballerup, Denmark). After 7 days on a given sodium diet, mice were either subjected to glomerular filtration rate (GFR) measurements, *in situ* biotinylation or killed thereafter for tissue and organ removal. Animals were anaesthetized by intraperitoneal injection of ketamine and xylazine (100 and 5 mg kg^−1^, respectively) (GRAEUB, Bayer Healthcare, Berlin, Germany). At the end of the experiments, animals were deeply anaesthetized with 50 mg (kg body wt)^−1^ pentobarbital i.p. (Sanofi, Toulouse, France). For *in vitro* studies (microdissection and immunoblotting), animals were studied 7 days after being placed on a given Na^+^ diet. Animal experiments were approved by the ethical committee of the University of Geneva and governmental authorities.

### Transcutaneous measurement of GFR (mGFR) in conscious mice

For GFR measurements, mice were anaesthetized with isoflurane, and a miniaturized imager device built from two light‐emitting diodes, a photodiode and a battery (Mannheim Pharma and Diagnostics, Mannheim, Germany) was mounted via double‐sided adhesive tape onto the shaved animal's neck. For the duration of the recording (1 h), each animal was conscious and kept in a single cage. Before the intravenous injection of 150 mg kg^−1^ fluorescein isothiocyanate (FITC)‐sinistrin (Mannheim Pharma and Diagnostics), the skin's background signal was recorded for 5 min. After removal of the imaging device, the data were analysed using MPD Lab software (Mannheim Pharma and Diagnostics). mGFR (μl min^−1^) was calculated from the decrease in fluorescence intensity over time (i.e. plasma half‐life of FITC‐sinistrin) using a two‐compartment model, the mouse body weight and an empirical conversion factor (Schreiber *et al*. 2012).

### Functional diuretic tests

On some of the animals that were fully adapted in metabolic cages, we performed diuretics response studies. Briefly, furosemide (10 mg kg^−1^), hydrochlorothiazide (50 mg kg^−1^), amiloride (5 mg kg^−1^) or benzamil (5 mg kg^−1^) were injected i.p. 7 days after the onset of various Na^+^ diets (Leviel *et al*. 2010; Morla *et al*. 2013; Al‐Qusairi *et al*. 2017). Urinary Na^+^ excretion was measured 24 h before and after diuretic administration, and 1, 4 or 6 h after the injection of furosemide, hydrochlorothiazide, and amiloride or benzamil, respectively. Results were expressed as μmol Na^+^ h^–1^.

### Microdissection of renal tubules

Briefly, the left kidneys of pentobarbital‐anaesthetized mice were infused via the abdominal aorta with an incubation solution [Hank's solution supplemented with 1 mm pyruvate, 0.1% bovine serum albumin (BSA), 0.5 mm MgCl_2_, 1 mm glutamine and 20 mm Hepes, pH 7.4] containing collagenase (337 UI mg^−1^, 0.18% w/v; Worthington, Biochemical Corp., Lakewood, NJ, USA). Kidneys were cut into small pieces which were incubated for 20–25 min at 32°C in an oxygenated incubation solution containing 0.1% collagenase. Renal tubules, i.e. proximal convoluted tubule (PCT), cTAL and cortical collecting duct (CCD), were microdissected under stereomicroscopic observation in an incubation solution supplemented with antiproteases (protease inhibitor cocktail tablets, Roche Diagnostics Corp., Indianapolis, IN, USA) at 4°C, as previously described (Gonin *et al*. 2001). For protein analysis by western blotting, each lane represents pools of microdissected tubules from two animals.

### Immunoblotting

Pools of 40–50 isolated tubules or 10 μg proteins extracted from kidney cortex were solubilized at 95°C for 5 min in Laemmli buffer and stored at −20°C until use. Proteins were subjected to SDS‐PAGE and transferred onto polyvinylidene difluoride membranes (Immobilon‐P; Millipore, Billerica, MA, USA). After blocking in TBS‐tween buffer (50 mm Tris base, 150 mm NaCl, 0.1% tween) containing 5% non‐fat dried milk, blots were successively incubated with specific antibodies. Antibodies used to detect Na^+^ transporters are listed in Table 1. Rabbit polyclonal antibodies raised against AMPK α‐subunit and acetyl‐CoA carboxylase (ACC; Cell Signaling Technology, Danvers, MA, USA) were diluted 1:1000 and those raised against phospho‐AMPK α‐subunit and phospho‐ACC (Cell Signaling) were diluted 1:500. Mouse monoclonal antibodies raised against E‐Cadherin (BD Biosciences Pharmingen, Franklin Lakes, NJ, USA) were diluted 1:1000, and those against β‐actin (Sigma, St Louis, MO, USA) were diluted 1:20,000. Anti‐β‐horseradish peroxidase‐conjugated secondary antibodies (BD Biosciences Pharmingen) diluted 1:20,000 (v/v) were used for detection of immunoreactive proteins by enhanced chemiluminescence (Millipore). Bands were quantified with ImageJ software. Results were expressed as the ratio of the densitometry of the band of interest to the loading control.

**Table 1:** Antibodies used for western blots

Name	Species	dilution	Supplier	Reference
NHE3	Mouse	1:500	Millipore	Biemesderfer *et al*. (1997)
NKCC2	Rabbit	1:1000	J Loffing	Wagner *et al*. (2008)
NCC	Rabbit	1:1000	J Loffing	Sorensen *et al*. (2013)
NCC‐pT53	Rabbit	1:1000	J Loffing	Sorensen *et al*. (2013)
α‐ENaC	Rabbit	1:1000	J Loffing	Wagner *et al*. (2008)
γ‐ENaC	Rabbit	1:1000	Stressmark	Klemens *et al*. (2017)
α‐Na,K‐ATPase	Rabbit	1:1000	E Feraille	Lourdel *et al*. (2005)

### *In situ* mouse kidney biotinylation

Renal plasma membranes were biotinylated *in situ* as described previously (Frindt *et al*. 2008), with some modifications. Kidneys were perfused with heparinized TBS for washing, then perfused with 0.5 mg ml^−1^ sulfosuccinimidyl‐2‐[biotinamido]ethyl‐1,3‐dithiopropionate (sulfo‐NHS‐biotin, Campbell Science, Rockville, IL, USA) at a rate of 1 ml min^−1^ for 30 min with a mini pump, through the descending aorta. The reaction was stopped and excess biotin was removed by further perfusion with TBS for 10 min. At the end of the perfusion, the left kidney was quickly removed, minced with a razor blade and homogenized with a tight‐fitting Dounce tissue grinder in 8 ml of lysis buffer containing (in mm) 250 sucrose, 10 triethanolamine HCl, 1.6 ethanolamine, 0.5 EDTA at pH 7.40 and 60 μl protease‐inhibitor cocktail (Sigma‐Aldrich). The homogenate was centrifuged at 1000 *g* for 10 min to separate intact tissue; supernatant was collected and frozen at –80°C for later use. The samples then centrifuged at 100,000 *g* for 2 h to sediment a total membrane pellet. This was resuspended in 2 ml of lysis buffer, aliquoted and frozen at −80°C for later analysis. Protein concentrations were measured with a micro BCA reagent kit (Pierce Chemical, Rockford, IL, USA). The isolation of biotinylated proteins used Neutravidin Ultralink beads (Pierce) as described previously for rat kidneys (Frindt & Palmer, 2015).

### Mathematical model

Model predictions of transepithelial sodium fluxes and oxygen consumption rates were obtained using a published model of transport along the superficial nephron of a rat (Layton *et al*. 2016). The model represents the tubules as cylinders, with axial flows and lateral fluxes across transcellular and paracellular pathways. It computes the transport rates of water and 15 solutes along the nephron, by considering the specific transporters (i.e. channels, exchangers, cotransporters and pumps) that are expressed on the apical and basolateral membranes in each segment. The underlying equations express conservation of mass and charge; they are solved at steady state to yield volume, concentrations and electric potential in the lumen and in each cell type, as a function of position. Interstitial concentrations are assumed to be known. Active O_2_ consumption is taken to be proportional to the Na, K‐ATPase transport rate.

The rat used in this model (taken to weigh 200 g and to have 60,000 nephrons) and the mice in our experiments have similar GFR per unit body weight [∼800 μl min^−1^ (100 g BW)^–1^]. Hence, to allow for a direct comparison between model predictions and experimental data, computed Na^+^ fluxes and O_2_ consumption rates are expressed in mmol h^−1^ (100 g BW)^–1^.

### Statistics

Results are given as means ± SE from *n* independent experiments. Comparisons between two groups were performed by unpaired Student's *t* test unless stated otherwise. Comparisons between more than two groups were performed by ANOVA and Dunnet's *t* test or by Kruskall–Wallis analysis of variance. A *P* value <0.05 was considered significant.

## Results

### Effect of dietary Na^+^ intake on physiological parameters

**Figure 1:**
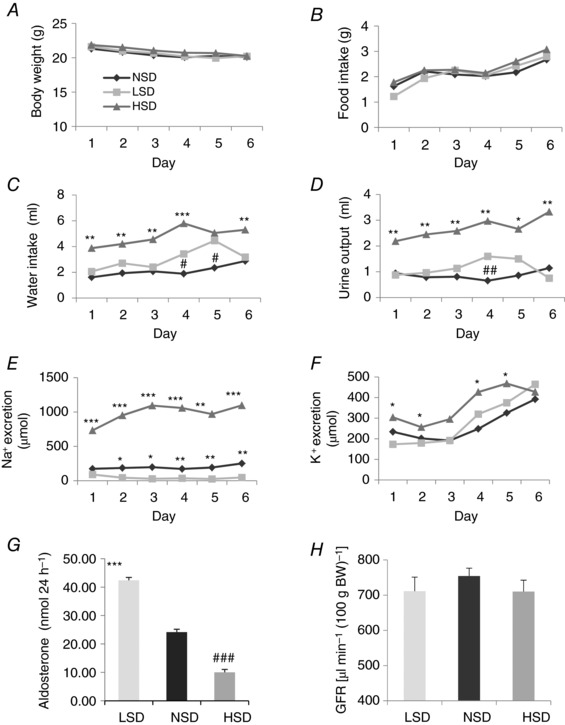
Effect of dietary Na^+^ intake on physiological parameters in mice *A*, body weight slightly decreased over the experimental period for all mice but without any detectable difference between LSD, NSD and HSD groups. *B*, food intake was similar in all groups under various dietary salt intakes. *C* and *D*, compared to LSD and NSD, HSD mice displayed a significant increase in water intake (*C*) and urinary volume (*D*). *E*, urinary Na^+^ excretion was highly increased under HSD and decreased under LSD as compared to NSD. *F*, urinary K^+^ excretion displayed a progressive rise following the increase in food intake, reflecting the adaptation of animals to the metabolic cage. K^+^ excretion was unchanged under LSD while it significantly increased under HSD. *G*, urinary aldosterone measured at day 7 revealed a 2.5‐fold increase in the LSD group and about a 2.5‐fold decrease in the HSD group, as compared to the NSD group. *H*, after 7 days, mGFR was similar in mice under various salt diets. Values are means ± SEM from 6–8 mice in each group. LSD and HSD were compared to NSD; ^*^ *P* < 0.05, ^**^ *P* < 0.01, ^***^ *P* < 0.001.

Mice were fed for 6 days with a low sodium diet (LSD, 0.01% Na^+^), a high sodium diet (HSD, 1.25% Na^+^) or a normal sodium diet (NSD, 0.18% Na^+^). All three groups displayed similar body weight and food intake (Fig. 1 *A* and *B*) as well as plasma [Na^+^] and [K^+^] (Table 2). As expected, under NSD, urinary Na^+^ excretion increased slightly in parallel with the progressive increase in food intake, reflecting adaptation to metabolic cages (Fig. 1 *E*). Water intake and urine output increased throughout the 6 days of HSD while urinary Na^+^ excretion reached a steady state after 3 days (Fig. 1 *C*–*E*). In contrast, water intake and urinary output were unchanged while urinary Na^+^ excretion significantly decreased during LSD (Fig. 1 *C*–*E*). Urinary K^+^ excretion displayed a progressive rise following the increase in food intake, reflecting adaptation to metabolic cages. K^+^ excretion was unchanged under LSD while it increased significantly under HSD (Fig. 1 *F*). Figure 1 *G* shows that, as expected, 24 h of urinary aldosterone, measured on day 6, increased strongly after LSD and was largely decreased after HSD, as compared to NSD. This finding is in agreement with previous studies showing that changes in dietary salt intake are accompanied by inverse changes in the activity of the renin–angiotensin–aldosterone system (Masilamani *et al*. 2002; Yang *et al*. 2008; Castrop *et al*. 2010). As previously reported in rats (Thomson *et al*. 2006), GFR measured as FITC‐sinistrin clearance after 7 days of diet was unchanged by LSD or HSD (Fig. 1 *H*). These results indicate that mice achieved steady‐state conditions.

**Table 2:** Plasma parameters

Diet	Na^+^ (mm)	K^+^ (mm)	Creatinine (mg dl^−1^)
LSD	155.3 ± 1.5 (7)	4.45 ± 0.13 (7)	0.15 ± 0.006 (7)
NSD	155 ± 0.4 (6)	4.5 ± 0.21 (6)	0.18 ± 0.013 (6)
HSD	154.4 ± 0.6 (8)	4.3 ± 0.12 (8)	0.17 ± 0.010 (8)

Results are expressed as means ± SEM from (n) animals.

### Effect of dietary Na^+^ on apical Na^+^ transporters and Na,K‐ATPase expression

**Figure 2:**
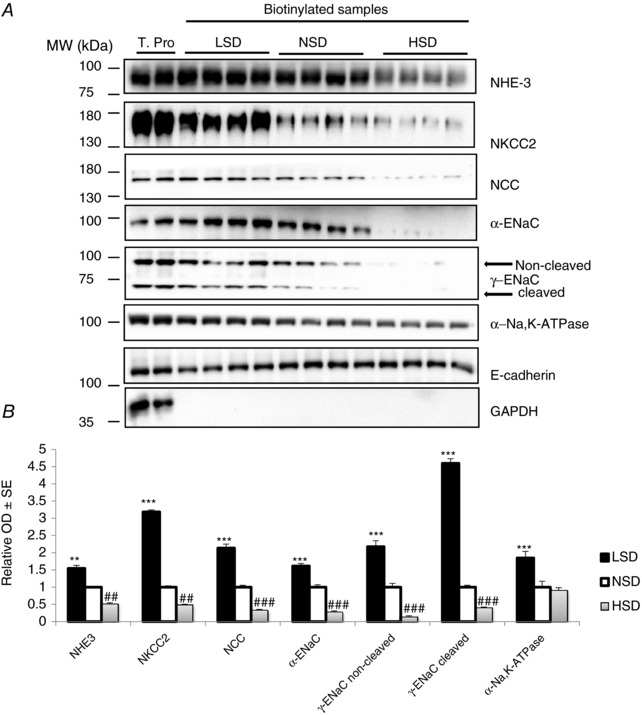
Cell‐surface expression of Na^+^ transporters and channel subunits under different Na^+^ diets After 7 days on different salt diets, cell‐surface proteins were biotinylated *in vivo* and biotinylated proteins were extracted by precipitation of streptavidin‐agarose beads and separated by SDS‐PAGE. *A*, representative western blotting experiments for the main Na^+^ transporters, namely NHE3, NKCC2, NCC, α‐ENaC, γ‐ENaC and α1‐NKA. The first two lanes are total proteins. *B*, bar graphs showing densitometric quantification of western blots. The plasma membrane protein E‐cadherin was taken as a loading control. Statistical analysis showed a significant decrease in the cell‐surface expression of apical NHE3, NKCC2, NCC, α‐ENaC, and the cleaved and non‐cleaved forms of γ‐ENaC in HSD mice. All these transporters in addition to α1‐NKA were over‐expressed in LSD animals. Values are means ± SEM from four mice in each group. LSD and HSD were compared to NSD; ^*^ *P* < 0.05, ^**^ *P* < 0.01, ^***^ *P* < 0.001.

We measured the cell‐surface expression of key Na^+^ transporters in response to different Na^+^ diets. Ion transporter activity is proportional to the number of molecules inserted into the plasma membrane and in contact with extracellular medium (Loffing *et al*. 2001). To assess the cell‐surface expression of kidney tubule Na^+^ transporters, we adapted the mice to the *in situ* biotinylation technique that had previously been applied to rats by Frindt *et al*. (2008). LSD significantly increased the cell‐surface abundance of NHE3, NKCC2, NCC, α‐ENaC, as well as both cleaved and non‐cleaved γ‐ENaC, whereas HSD significantly decreased the apical surface abundance of all these ion transporter proteins (Fig. 2). These results suggest that LSD increased while HSD decreased overall apical Na^+^ transporter abundance along the mouse renal tubule.

**Figure 3:**
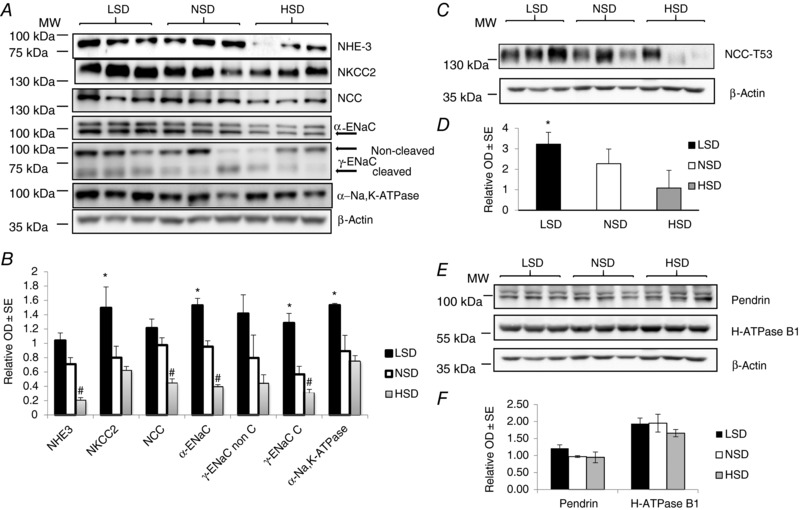
Na^+^ transporter abundance under different Na^+^ diets in total kidney lysate After 7 days on a given salt diet, kidneys were harvested and total proteins were extracted and separated by SDS‐PAGE. *A*, western blotting experiments for the main tubular Na^+^ transporters, namely NHE3, NKCC2, NCC, α‐ENaC, γ‐ENaC and NKA. *B*, bar graph showing densitometric quantification of western blots.  β‐Actin was used as a loading control. Statistical analysis showed a significant decrease in the expression of NHE3, NCC, α‐ENaC and the cleaved form of γ‐ENaC in kidneys from mice under HSD. Kidneys from mice under LSD displayed a significant increase in the expression of NKCC2, α‐ENaC, NKA and the cleaved form of γ‐ENaC. *C*, western blotting experiment for NCC‐pT53. *D*, bar graph showing densitometric quantification of western blot.  β‐Actin was used as a loading control. *E*, western blotting experiments for Pendrin and H‐ATPase B1 subunit are shown. *F*, bar graph showing densitometric quantification of Western blot. Values are means ± SEM from three mice in each group. LSD and HSD were compared to NSD; ^#^ or ^*^ *P* < 0.05, ^##^ or ^**^ *P* < 0.01, ^####^ or ^***^ *P* < 0.001.

We then assessed the total abundance of kidney tubule Na^+^ transporters by western blotting in kidney cortex extracts. LSD compared to NSD significantly increased the total protein abundance of NHE3, NKCC2, α‐ENaC and cleaved γ‐ENaC (Fig. 3). In contrast to its cell‐surface abundance, the total abundance of NCC was not significantly increased. HSD, by contrast, significantly decreased the total expression levels of NHE3, NCC, α‐ENaC and cleaved γ‐ENaC. The abundance of NKCC2 and the non‐cleaved form of γ‐ENaC was not significantly changed under HSD (Fig. 3 *A* and *B*). These results indicate that dietary sodium‐induced variations in the cell‐surface abundance of the major kidney tubule Na^+^ transporters are not necessarily associated with parallel changes in their total abundance. We also measured the phosphorylation of NCC on threonine 53, a surrogate of its activity. NCC phosphorylation was, as expected, increased under LSD and decreased under HSD (Fig. 3 *C* and *D*). In addition, we assessed the effect of dietary salt on the expression of H‐ATPase and Pendrin, the major apical acid–base transporters of α‐ and β‐intercalated cells, respectively. Pendrin and H‐ATPase abundance was not significantly altered by changes in dietary salt intake (Fig. 3 *E* and *F*).

The ubiquitous Na,K‐ATPase (dietary NKA) located along the basolateral membranes of tubular epithelial cells is the major pathway for Na^+^ extrusion. Expression of the NKA α1‐subunit, the almost exclusive kidney isoform (Féraille & Doucet, 2001), increased in both cell‐surface and total kidney cortex protein extracts under LSD but remained unchanged under HSD (Figs 2 and 3). Therefore, the abundance of NKA increases in parallel with that of apical Na^+^ transporters under LSD, but it does not follow the general decrease in apical Na^+^ transporter abundance observed under HSD. This pattern is at variance with the observed coordinated regulation of apical NHE3 and basolateral Na,K‐ATPase in response to dopamine or angiotensin II in the proximal convoluted tubule (Féraille & Doucet, 2001).

### Mathematical modelling of Na^+^ transport along the nephron

**Figure 4:**
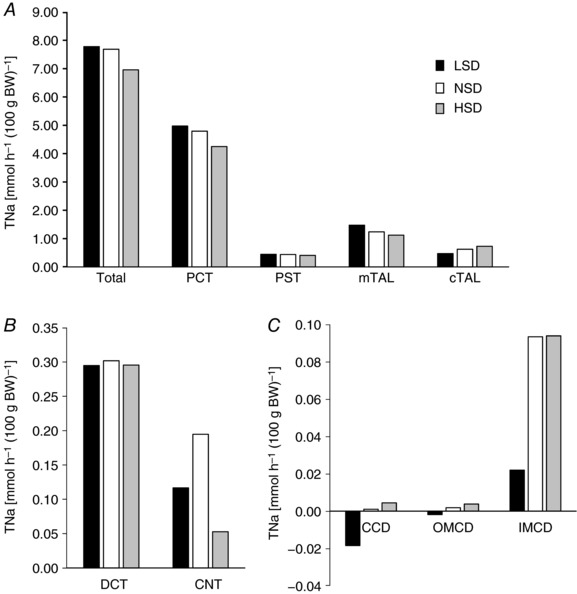
Mathematical modelling of tubular Na^+^ transport under different salt diets Using an *in silico* model of Na^+^ transport along the rat renal tubules, we computed the rates of Na^+^ transport (TNa) along the nephron. Results account for the fractional changes in cell‐surface expression as listed in Table 3 (case 1). Shown is the predicted TNa [mmol h^−1^ (100 g body weight)^–1^] for: *A*, the whole nephron, PCT, PST, cTAL and mTAL; *B*, DCT and CNT; *C*, CCD, OMCD and IMCD. Relative to NSD, Na^+^ reabsorption under LSD is predicted to slightly increase in PCTs, PSTs and mTALs and to decrease in cTALs, DCTs, CNTs and all CD segments. Conversely, Na^+^ reabsorption under HSD is predicted to decrease in PCTs, PSTs, mTALs, DCTs and CNTs, and to increase in cTALs and all CD segments.

We assessed how tubular Na^+^ transport (TNa) was redistributed along the nephron under LSD or HSD by running simulations with a previously described mathematical model (Layton *et al*. 2016). Since the model applies to a rat kidney, we focused on the relative changes in transepithelial transport and urinary excretion. Results are thus expressed. Results are given per unit body weight as described above. In these simulations, we accounted for the measured fractional changes in cell‐surface expression levels when available or total abundance (see Figs 2 and 3), as summarized in Table 3. The predicted and measured diet‐induced variations in the urinary excretion of Na^+^ and K^+^ were in good agreement overall (Table 4). The computed value of overall Na^+^ transport increased very slightly (by 1%) under LSD and decreased by 9% under HSD (Fig. 4 *A*). The model predicts that relative to NSD, Na^+^ reabsorption under LSD is increased in the proximal nephron from the PCT to the proximal straight tubule (PST) and the mTAL (Fig. 4 *A*), and reduced in the segments downstream, including the cTAL, the DCT, the CNT and the entire CD (Fig. 4 *B* and *C*). TNa increases the most (in both absolute and relative terms) in the mTAL, given the 3.2‐fold increase in the cell‐surface expression of NKCC2. Downstream, TNa decreases despite the increase in transporter expression, owing to diminished Na^+^ delivery. In particular, net Na^+^ secretion is predicted in the CCD and outer medullary collecting duct (OMCD) under LSD (Fig. 4 *C*) because low Na^+^ delivery to that segment strongly stimulates paracellular Na^+^ secretion, which more than counterbalances transcellular Na^+^ reabsorption. Thus, our results suggest that TNa is governed not only by Na^+^ transporter abundance but also by Na^+^ delivery to a given segment.

**Table 3:** Fractional changes in transporter expression assumed in model simulations

Transporter expression relative to NSD	LSD	HSD
NHE3 in PT	+54%	−48%
Na,K‐ATPase in PT	+85% (+104%)	−9% (+55%)
NKCC2 in mTAL	+219%	−51%
Na,K‐ATPase in mTAL	+85%	−9%
NKCC2 in cTAL	+219% (+32%)	−51% (−18%)
Na,K‐ATPase in cTAL	+85% (+9%)	−9% (+4%)
NCC in DCT (Phospho T53)	+42%	−52%
Na,K‐ATPase in DCT	+85%	−9%
γ‐ENaC in CCD	+118% (–)	−85% (–)
Na,K‐ATPase in CCD	+85% (+92%)	−9% (+279%)
γ‐ENaC in CNT, OMCD, IMCD	+118%	−85%
Na,K‐ATPase in CNT, OMCD, IMCD	+85%	−9%
Pendrin in CNT and CCD	+25%	−2%
Apical H‐ATPase in CNT, CCD, OMCD	–	−15%

PT, proximal tubule; mTAL/cTAL, medullary/cortical thick ascending limb of Henle; DCT, distal convoluted tubule; CNT, connecting tubule; CCD, cortical collecting duct; OMCD/IMCD, outer/inner medullary collecting duct. Fractional changes were measured using an *in situ* biotinylation technique or total protein extracts (Phospho‐NCC, Pendrin and H‐ATPase). Values in parentheses correspond to segment‐specific results (case 2), as opposed to whole‐kidney values.

**Table 4:** Predicted *vs*. measured fractional changes in solute excretion

		LSD‐to‐NSD ratio	HSD‐to‐NSD ratio
Na^+^ excretion	Experimental	0.11	4.0
	Model case 1	0.11	8.4
	Model case 2	0.12	9.3
K^+^ excretion	Experimental	0.71	1.2
	Model case 1	0.20	1.5
	Model case 2	0.21	1.7

Experimental results correspond to day 7 after the onset of the low‐ or high‐sodium diet. Model case 1 accounts only for whole‐kidney changes in transporter expression. Model case 2 also considers segment‐specific changes in transporter expression. See text for details.

Conversely, the model predicts that, relative to NSD, Na^+^ reabsorption under HSD diminishes all the way to the connecting tubule, except for the cTAL (Fig. 4 *A*), and increases in the CCD and OMCD (Fig. 4 *B* and *C*). The largest TNa reduction occurs in the PT, as a result of the 2‐fold reduction in NHE3 expression (Table 3). By contrast, TNa increases slightly in the collecting duct, despite the decrease in ENaC and NKA membrane expression: indeed, higher Na^+^ delivery reduces paracellular secretion along that segment.

### Effect of dietary Na^+^ on expression of Na^+^ transporters in microdissected tubules

**Figure 5:**
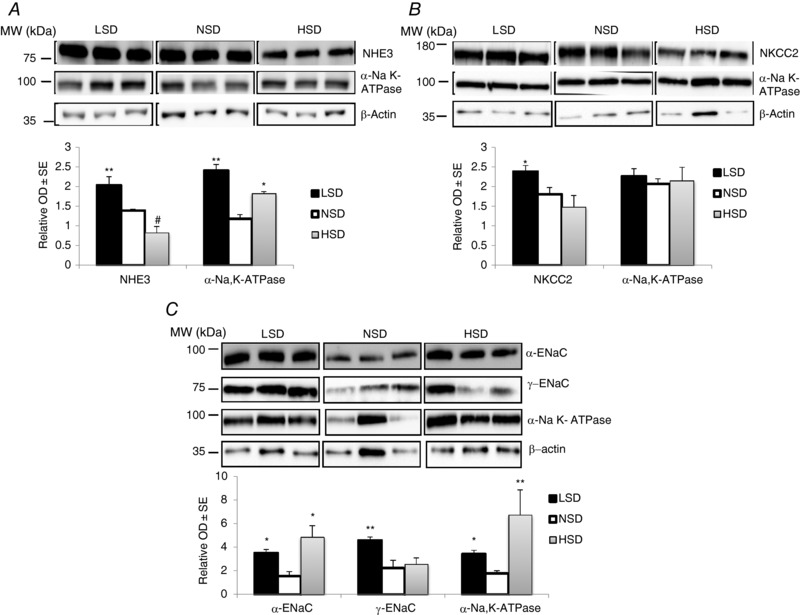
The effect of different salt diets on the expression of Na^+^ transporters in microdissected tubules After 7 days on different salt diets, isolated well‐defined nephron segments were microdissected from collagenase‐treated kidneys. Pools of 100 tubules from two different animals were lysed and proteins were separated by SDS‐PAGE followed by western blotting and densitometric quantification. *A*, abundance of NHE3 and α1‐NKA increased in PCTs from mice under LSD. In PCTs from mice under HSD, NHE3 expression decreased while α1‐NKA abundance increased. *B*, NKCC2 expression significantly increased and slightly decreased in TALs from mice under LSD or HSD, respectively. α1‐NKA expression was unchanged. *C*, in CCDs, α‐ENaC and NKA expression were significantly increased in mice under both LSD and HSD. However, γ‐ENaC abundance was increased only in mice under LSD. Each lane represents protein level from tubules pooled from two independent animals. Values are means ± SEM from three pools from six mice in each group (microdissected tubules from two animals are pooled in the same lane). LSD and HSD were compared to NSD; ^*^ *P* < 0.05, ^**^ *P* < 0.01, ^***^ *P* < 0.001.

We dissected PCT, cTAL and CCD from collagenase‐treated kidney cortices. In microdissected PCTs, NHE3 expression increased under LSD while it decreased under HSD, as compared to NSD. These results are similar to those observed in cell‐surface protein extracts and whole kidney cortex homogenates. By contrast, NKA α1‐subunit protein abundance increased both in LSD and in HSD (Fig. 5 *A*). In the cTAL, NKCC2 expression increased under LSD whereas α1‐NKA abundance was unchanged, as compared to NSD. These results match those obtained in cell‐surface protein extracts and whole kidney cortex homogenates. On the other hand, HSD altered neither NKCC2 nor α1‐NKA expression levels in cTAL (Fig. 5 *B*). This observation is in agreement with results obtained in whole kidney cortex but at variance with the observed decrease in NKCC2 abundance in cell‐surface protein extracts. This result in cTAL suggests that NKCC2 abundance is largely decreased in mTAL under HSD. We also assessed the changes in the expression levels of ENaC subunits and NKA in the CCD, an aldosterone‐sensitive tubular segment where the fine‐tuning of sodium reabsorption occurs. In agreement with results obtained in cell‐surface and total cortex protein extracts, LSD increased the abundance of α‐ENaC and γ‐ENaC subunits as well as that of α1‐NKA proteins. The amount of proteins loaded on the gel was not sufficient to detect the cleaved form of γ‐ENaC. On the other hand, HSD increased both α‐ENaC and α1‐NKA protein expression, whereas γ‐ENaC abundance was unchanged (Fig. 5 *C*). These patterns are at variance with the observed decrease in both α‐ENaC and γ‐ENaC in both cell‐surface and total cortex protein extracts. This apparent discrepancy probably reflects the differential control of ENaC subunit abundance in the late DCT and CNT, as compared to the CD. We were not able to hand‐microdissect a large enough number of DCTs and CNTs that would allow the detection of NCC and ENaC subunits by western blotting. Taken together, our results indicate that dietary Na^+^ intake influences Na^+^ transporter expression in a segment‐specific pattern that is, at least partly, independent of variations in aldosterone secretion.

### Mathematical modelling of segment‐specific Na^+^ transport

**Figure 6:**
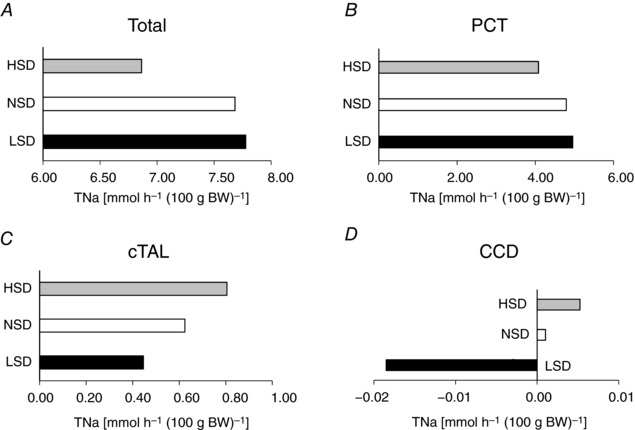
Mathematical modelling of Na^+^ transport in isolated nephron segments under different salt diets TNa was determined accounting for the specific changes in transporter expression in the PCT, cTAL and CCD (Table 3, case 2). Results are given for the whole nephron (*A*), PCT (*B*), cTAL (*C*) and CCD (*D*). Model predictions under LSD are very similar to those shown in Fig. 3. Relative to NSD, NaK expression under HSD was higher in the PCT, cTAL and CCD; whole‐kidney TNa was nevertheless lower, because the decrease in apical Na^+^ transporter expression was the dominant factor.

We then performed another set of simulations to account for the segment‐specific, fractional changes in apical Na^+^ transporter and NKA expression in the PCT, cTAL and CCD. Model assumptions for this scenario (denoted case 2) are also shown in Table 3. Compared to the results obtained using only whole‐kidney data (case 1, see above), the computed value of whole kidney TNa remains unchanged under LSD, and is 1% lower under HSD. The latter result may appear counter‐intuitive: since Na,K‐ATPase expression in the PCT, cTAL and CCD is higher in case 2 than in case 1 under HSD, overall Na^+^ reabsorption is expected to increase rather than to decrease. TNa is indeed higher in the cTAL and CCD (case 2 *vs*. case 1), but it is 4% lower in the PCT (Fig. 6), because of counterbalancing effects. [According to our simulations, when NHE3 expression is reduced by half, increased NaK activity in the PCT lowers the intracellular concentration of Na^+^ such that basolateral Na^+^ (and HCO_3_^–^) entry via Na^+^‐dependent Cl^–^/HCO_3_^–^ exchangers (NDCBE) is strongly stimulated. The resulting increase in intracellular pH in turn reduces apical Na^+^/H^+^ exchange across NHE3, lowers the transepithelial voltage and thereby reduces paracellular Na^+^ reabsorption as well.]

Overall our results suggest that, in the absence of changes in the delivered load, the major determinant of TNa is abundance of the apical Na^+^ transporter. Given a fixed load, Na^+^ reabsorption is predicted to decrease (relative to NSD) in segments where the expression of apical transporters decreases, even when the expression of basolateral NKA increases concomitantly. This is exemplified in the PCT where decreased Na^+^ reabsorption under HSD is explained by decreased NHE3 expression despite increased NKA expression (Fig. 6).

### Functional assessment of nephron segment‐specific Na^+^ transporters

**Figure 7:**
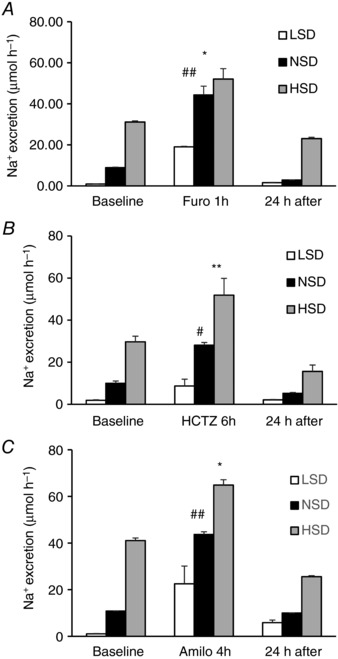
Functional assessment of renal sodium reabsorption in response to diuretics After 7 days on different salt diets, mice were injected i.p. with a single dose of (*A*) furosemide, which inhibits NKCC2 in the TAL, (*B*) hydrocholorothiazide, which inhibits NCC in the DCT, or (*C*) amiloride, which inhibits ENaC in the CNT–CD. The left bars show steady‐state Na^+^ excretion 24 h before diuretic administration, the middle bars show the natriuretic response to diuretics (1 h for furosemide, 6 h for hydrochlorothiazide and 4 h for amiloride), and the right bars show Na^+^ excretion during the 24 h recovery period. Values are means ± SEM from four mice in each group. LSD and HSD were compared to NSD; ^*^ *P* < 0.05, ^**^ *P* < 0.01.

Diuretics promote natriuresis and diuresis by inhibiting tubular Na^+^ reabsorption at specific nephron sites. Loop diuretics inhibit NKCC2 (Velázquez & Wright, 1986) and thus Na^+^ reabsorption in the TAL (Greger, 1985). The effect of furosemide on natriuresis measured 1 h after the injection was lower under both LSD and HSD, as compared to NSD (Fig. 7 *A*). This decreased furosemide‐induced natriuresis is consistent with decreased Na^+^ delivery to the TAL under LSD and decreased NKCC2 abundance under HSD.

Thiazides decrease Na^+^ reabsorption mostly by blocking NCC in the DCT (Velázquez & Wright, 1986). In response to hydrochlorothiazide, Na^+^ excretion was slightly higher under HSD and lower under LSD, as compared to NSD (Fig. 7 *B*). The higher absolute Na^+^ excretion observed under HSD might be explained by decreased ENaC abundance in the connecting tubule and collecting duct (see Fig. 2). This may impair the compensation of diuretic‐induced natriuresis via increased Na^+^ reabsorption by the CNT and CD, as suggested by model predictions (results not shown). Conversely, the decreased natriuretic response observed under LSD may at least in part also rely on increased ENaC abundance (see Fig. 2), leading to enhanced Na^+^ reabsorption downstream from the DCT.

In the aldosterone‐sensitive distal nephron [i.e. the distal convoluted tubule (DCT2), connecting tubule and collecting duct], the amiloride‐sensitive epithelial Na^+^ channel ENaC, consisting of α‐, β‐ and γ‐subunits, mediates Na^+^ uptake across the apical plasma membrane of principal cells (Loffing & Kaissling, 2003). Figure 7 *C* shows that the effects of amiloride on Na^+^ excretion were almost similar under the three different dietary sodium intakes, pointing to the strong impact of sodium delivery which counteracts the effects of variations in ENaC abundance under LSD and HSD. Control experiments indicate that the observed natriuresis and diuresis in response to diuretics is not affected by the intraperitoneal infusion of the vehicle (0.9% NaCl). Increased natriuresis in response to amiloride under HSD (relative to NSD) may occur in part because net Na^+^ reabsorption is higher along the collecting duct in the absence of inhibitors, as predicted by the model (see Figs 4 and 6). In contrast, the reduced response to amiloride observed under LSD may stem from increased Na^+^ delivery to the collecting duct, which lowers paracellular Na^+^ secretion along the upper portion of this segment, according to our computations.

### Regulation of AMPK activity by dietary Na^+^ intake

**Figure 8:**
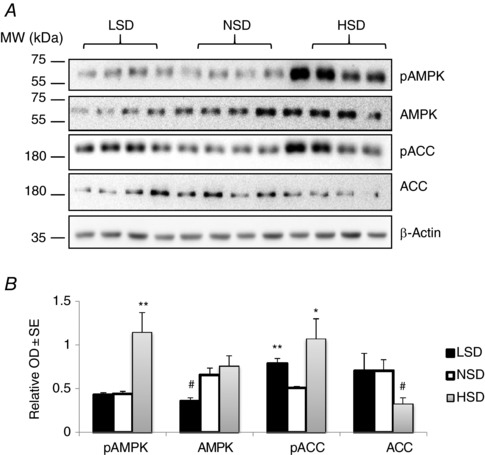
Regulation of AMPK expression and activity by dietary salt in total kidney lysates After 7 days on different salt diets, kidneys were harvested and total proteins were extracted and separated by SDS‐PAGE. *A*, western blotting experiments for the 5′ AMP‐activated protein kinase (AMPK) pathway including total and phosphorylated AMPK (pAMPK) in addition to total (ACC) and phosphorylated acetyl‐CoA carboxylase (pACC). Representative immunoblots from three animals from each group are shown. *B*, bar graphs showing densitometric quantification of western blots.  β‐Actin was used as a loading control. Statistical analysis showed a significant increase in pAMPK and pACC levels in kidneys from mice under HSD. LSD induced a significant reduction in total AMPK abundance but increased the phosphorylation level of ACC. Values are means ± SEM from six mice in each group. LSD and HSD were compared to NSD; ^*^ *P* < 0.05, ^**^ *P* < 0.01, ^***^ *P* < 0.001.

**Figure 9:**
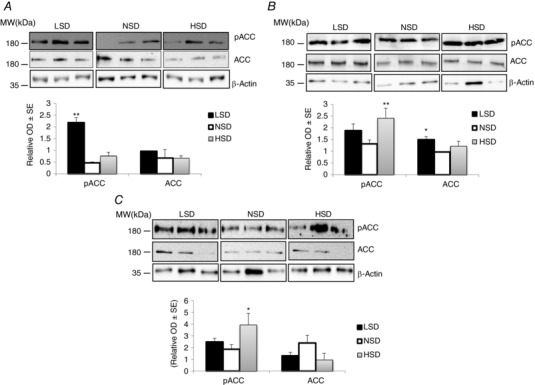
Regulation of AMPK activity by dietary salt in microdissected tubules After 7 days on different salt diets, isolated well‐defined nephron segments were microdissected from collagenase‐treated kidneys. Pools of 100 tubules from two different animals were lysed and proteins were separated by SDS‐PAGE followed by western blotting and densitometric quantification. A representative western blot for total (ACC) and phosphorylated ACC (pACC) is shown in upper panels. Bar graphs show densitometric quantification of western blots in lower panels.  β‐Actin was used as a loading control. *A*, both HSD and LSD increased the abundance of pACC without changing the total form of this protein in PCTs. *B*, in cTALs, only HSD increased pACC levels. *C*, in CCDs, pACC levels were significantly increased in response to HSD. Each lane represents protein level from tubules pooled from two independent animals. Values are means ± SEM from three pools from six mice in each group (microdissected tubules from two animals are pooled in the same lane). LSD and HSD were compared to NSD; ^*^ *P* < 0.05, ^**^ *P* < 0.01, ^***^ *P* < 0.001.

Increasing transcellular Na^+^ reabsorption by renal epithelial cells augments ATP consumption and thus results in an increased cytosolic AMP‐to‐ATP ratio that may activate AMPK, as previously suggested in γ‐ENaC‐TetOn‐mCCD cells (Wang *et al*. 2014). We estimated the activity of AMPK in total kidney extracts from mice subjected to various salt diets. Under HSD the phosphorylation levels of AMPK α‐subunit and its substrate ACC were significantly increased, reflecting an increase in AMPK activity (Fig. 8). To further assess the changes in AMPK activity along the kidney tubule, we microdissected specific nephron segments (PCT, cTAL, CCD) and measured the amounts of total and phosphorylated ACC. Under HSD, phosphorylated ACC (pACC) was significantly increased in cTALs and CCDs (Fig. 9 *A*–*C*). LSD, on the other hand, increased pACC in PCTs (Fig. 9 *A*) whereas it remained unchanged in cTALs and CCDs (Fig. 9 *B* and *C*). These changes in AMPK activity were positively correlated with estimated increases in active Na^+^ reabsorption in PCTs (see Fig. 6).

### Modelling of segment‐specific O_2_ consumption and tubular Na^+^ transport efficiency

**Figure 10:**
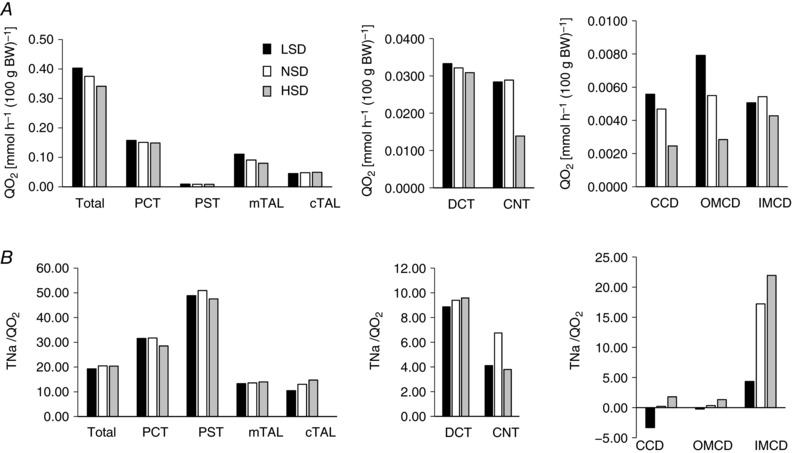
Mathematical modelling of oxygen consumption and the efficiency of Na^+^ transport under different salt diets The rate of O_2_ consumption (QO_2_; *A*) and the efficiency of Na^+^ transport (TNa/QO_2_; *B*) were computed using an *in silico* model of Na^+^ transport along the rat nephron. Under LSD, total QO_2_ was predicted to increase slightly. Relative to NSD, it was higher in PCTs, mTALs, DCTs, CCDs and OMCDs, and lower in cTALs and IMCDs. Conversely, under HSD, total QO_2_ was predicted to decrease slightly, particularly as a result of a significant decrease in mTALs and CNTs. *B*, under LSD, total TNa/QO_2_ slightly decreased, relative to NSD. The TNa/QO_2_ decrease was particularly pronounced in all CD segments. Under HSD, total TNa/QO_2_ slightly decreased as well. TNa/QO_2_ was predicted to decrease in PCTs, PSTs and CNTs and to increase in cTALs and all CD segments.

Tubular Na^+^ reabsorption is an active process that consumes both ATP and O_2_. Simulations of Na^+^ transport in specific nephron segments indicate that under HSD, O_2_ consumption rose in the PT and cTAL, while it decreased slightly in the DCT and more substantially in the CNT and CD. Under LSD, O_2_ consumption rose in the PT, mTAL, DCT, CCD and OMCD but slightly decreased in cTAL and inner medullary collecting duct (IMCD) (Fig. 10 *A*). Our model also suggests that the overall efficiency of Na^+^ transport, as reflected by the whole nephron ratio of TNa to active O_2_ consumption (denoted TNa/QO_2_), decreased slightly under both diets. As shown in Fig. 10 *B*, TNa/QO_2_ was highest in PCT and PST, where paracellular and transcellular transport contribute almost equally to Na^+^ reabsorption; TNa/QO_2_ was significantly lower in downstream segments, where Na^+^ backleak across the paracellular pathway can partially counteract transcellular Na^+^ reabsorption. In HSD, a significant fraction of Na^+^ transport was shifted from the PCT to downstream segments, hence modifying transport efficiency, which rose slightly in cTAL, DCT and CD but decreased in CNT. In LSD, the increase in NHE3 abundance enhanced the contribution of the transcellular pathway relative to that of the paracellular pathway in the PT, which is why TNa/QO_2_ was predicted to decrease in this case as well. Under this condition, the efficiency of Na^+^ transport was decreased (albeit with variable intensity) all along the downstream tubule segments. Overall, these results suggest that changes in dietary salt intake elicit a redistribution of O_2_ consumption and variations in TNa/QO_2_ along the kidney tubule.

## Discussion

The objective of this study was to revisit the patterns of the kidneys’ adaptive response to dietary Na^+^ changes using a combination of measurements of physiological parameters and Na^+^ transporter protein abundance, functional response to diuretic tests and mathematical modelling. Indeed, previous studies analysed separately the effect of dietary Na^+^ on either GFR or Na^+^ transporter abundance but, to our knowledge, none of them performed a complete analysis of the effects of dietary Na^+^ content. Our results show that plasma Na^+^ levels and GFR did not change after 1 week on a diet containing various amounts of Na^+^. This means that the filtered Na^+^ load remained similar under all conditions studied. In addition, previous studies have shown that systemic BP does not change under these short periods of altered diet (Holtbäck *et al*. 1993; Meneton *et al*. 2005; Brochu *et al*. 2013; Walkowska *et al*. 2017). Therefore, alterations in natriuresis were only due to changes in Na^+^ handling along the renal tubule.

Our results show that under LSD, the mouse kidney reduces urinary Na^+^ excretion, which is associated with increased expression of NHE3 in PTs, NKCC2 in TALs, NCC in DCTs and ENaC subunits in CNTs/CDs. A previous study using the same methodology indicated that in rats, LSD for 1 week did not alter either total and cell‐surface abundance of NHE3 or NKCC2, revealing species differences. In contrast, cell‐surface NCC and both total and cell‐surface abundance of ENaC subunits were increased in both mice (our study) and rats (Frindt & Palmer, 2009). As previously shown in rats (Frindt & Palmer, 2009), the increase in ENaC subunit abundance observed in our mouse study is probably dependent on increased aldosterone levels. Our simulations of tubular Na^+^ transport showed that Na^+^ reabsorption was increased in the PT, as also reflected by a rise in AMPK activity, leading to decreased downstream Na^+^ delivery and Na^+^ reabsorption. This interpretation is confirmed by the reduced natriuretic response to diuretics targeting the TAL and DCT.

The present study demonstrates that the mouse kidney adapts to a moderate HSD by increasing urinary Na^+^ and Cl^−^ excretion, which is associated with decreased cell‐surface abundance of NHE3 in PTs, NKCC2 in TALs, NCC in DCTs and ENaC subunits in CNTs/CDs. At variance with our findings in mice, Frindt & Palmer (2009) found that total and cell‐surface NHE3, NKCC2 and NCC abundance were not altered in rats fed with an HSD (5% NaCl) for 1 week. These differences may stem from different protocols (5% *vs*. 3% NaCl) and species‐specific effects. However, total and cell‐surface abundance of α‐ and γ‐ENaC were similarly decreased in both mice and rats (Frindt & Palmer, 2009). Our simulations of tubular Na^+^ transport indicate that decreased Na^+^ reabsorption by the kidney under HSD mostly results from decreased Na^+^ reabsorption by the PT. This adaptation of Na^+^ transport in the PT increases Na^+^ delivery and may lead to increased metabolic work in some downstream segments. On the whole, the calculated efficiency of whole‐nephron tubular Na^+^ transport was only slightly decreased. Alteration of the efficiency of tubular Na^+^ transport has been also reported under conditions that alter NO synthesis, such as treatment with an NO synthase blocker or 5/6 nephrectomy (Epstein *et al*. 1994; Welch *et al*. 2005). It should be mentioned that more subtle changes occur at the level of specific nephron segments such as the CCD, where increased expression of α‐ENaC and Na,K‐ATPase α1‐subunit are observed. These alterations cannot be due to increased aldosterone levels and imply that regulation of Na^+^ transport by the collecting duct occurs via both aldosterone‐dependent and ‐independent mechanisms that may rely on secretion of paracrine factors such as the recently described α‐ketoglutarate pathway (Grimm *et al*. 2015).

Whether NHE3 is regulated by dietary Na^+^ remained controversial. In one study, NHE3 expression was found to increase after salt restriction (Fisher *et al*. 2001). Other studies showed no change in NHE3 abundance in response to alterations in dietary Na^+^ (Masilamani *et al*. 2002; Thomson *et al*. 2006; Yang *et al*. 2008), or else found that HSD induces a redistribution of NHE3 from the top to the base of the microvilli (Yang *et al*. 2008). Our results indicate that under steady‐state conditions, HSD decreases both NHE3 expression and Na^+^ transport along the mouse PT while LSD results in opposite changes. In accordance with our results, Thomson *et al*. (2006) reported that in rats, salt loading for 7 days decreased Na^+^ transport in the PT.

Some studies showed no changes in the abundance of NKCC2 with HSD (Ecelbarger *et al*. 1996; Yang *et al*. 2008), while others found increased NKCC2 abundance in response to chronic saline loading (Song *et al*. 2004). Loffing *et al*. (2000) found that in rats HSD decreased α‐ENaC expression and induced a redistribution of β‐ENaC and γ‐ENaC from apical to intracellular pools. We found that HSD decreased the whole‐kidney expression of all ENaC subunits, whereas in microdissected CCDs the expression of α‐ENaC increased but not that of γ‐ENaC. This observation implies that ENaC expression diminishes in the late DCT and CNT but not in the CCD.

Tubular Na^+^ reabsorption is the major source of ATP and oxygen consumption in the kidney (Lassen & Thaysen, 1961; Brodwall & Laake, 1964; Mandel, 1986), and AMPK activity may reflect the intensity of active Na^+^ handling (Hallows *et al*. 2000). We have previously shown that enhanced Na^+^ transport in cultured CD principal cells (Wang *et al*. 2014) increased AMPK α‐subunit and ACC phosphorylation (Ha *et al*. 1994), indicating AMPK activation. Hallows *et al*. (2000) suggested that AMPK may link cellular metabolism and ion transport. In the present study, we found a globally positive relationship between estimated Na^+^ transport and AMPK activity in isolated nephron segments. Therefore, AMPK activity as estimated by phosphorylated ACC may represent an indication of increased tubular Na^+^ transport.

In conclusion, our results show that in response to variations in dietary salt intake, changes in tubular Na^+^ transport occur via both regulations of transporter expression as well as Na^+^ delivery. This study suggests that Na^+^ transport along a given segment is strongly impacted by Na^+^ delivery to that segment. In addition, our results indicate that variations in dietary Na^+^ induce a redistribution of tubular Na^+^ transport in the mouse kidney. These findings should be further confirmed in humans.

## Additional information

### Author contributions

KU: designed research studies, conducted experiments, acquired data, analysed data, and wrote the manuscript. AA: designed research studies, conducted experiments, acquired data. IR: conducted experiments, acquired data, analysed data. ED: conducted experiments, acquired data. MM: performed aldosterone level measurements. CB: performed GFR measurements and metabolic cage experiments. CAW: designed *in vivo* research studies. JL: provided material, designed experiments, analysed data. AE: designed research studies, performed mathematical model simulations, analysed data, and wrote manuscript. EF: designed research studies, conducted experiments, analysed data, and wrote manuscript. All authors read and approved the final manuscript.

### Funding

This work was supported by the National Centre of Competence in Research Kidney control of homeostasis and a Swiss National Science Foundation grant 31003A_156736/1 to EF, 310030_143929/1 to JL and 31003A_155959 to CAW. KU received funding within the framework of IKPP2 from the European Union's Seventh Framework Programme for research, technological development and demonstration under grant agreement no. 608847.

## References

[tjp12627-bib-0001] Al‐Qusairi L , Basquin D , Roy A , Rajaram RD , Maillard MP , Subramanya AR & Staub O (2017). Renal tubular ubiquitin‐protein ligase NEDD4‐2 is required for renal adaptation during long‐term potassium depletion. J Am Soc Nephrol 28, 2431–2442. 28289184 10.1681/ASN.2016070732 PMC5533229

[tjp12627-bib-0002] Biemesderfer D , Rutherford PA , Nagy T , Pizzonia JH , Abu‐Alfa AK & Aronson PS (1997). Monoclonal antibodies for high‐resolution localization of NHE3 in adult and neonatal rat kidney. Am J Physiol 273, F289–F299. 9277590 10.1152/ajprenal.1997.273.2.F289

[tjp12627-bib-0003] Boero R , Pignataro A & Quarello F (2002). Salt intake and kidney disease. J Nephrol 15, 225–229. 12113591

[tjp12627-bib-0004] Brochu I , Houde M , Desbiens L , Simard E , Gobeil F , Semaan W , Bkaily G & D'Orléans‐Juste P (2013). High salt‐induced hypertension in B2 knockout mice is corrected by the ETA antagonist, A127722. Br J Pharmacol 170, 266–277. 23713522 10.1111/bph.12259 PMC3834752

[tjp12627-bib-0005] Brodwall EK & Laake H (1964). The relation between oxygen consumption and transport of sodium in the human kidney. Scand J Clin Lab Invest 16, 281–286. 14164780

[tjp12627-bib-0006] Canessa CM , Schild L , Buell G , Thorens B , Gautschi I , Horisberger JD & Rossier BC (1994). Amiloride‐sensitive epithelial Na^+^ channel is made of three homologous subunits. Nature 367, 463–467. 8107805 10.1038/367463a0

[tjp12627-bib-0007] Castrop H , Höcherl K , Kurtz A , Schweda F , Todorov V & Wagner C (2010). Physiology of kidney renin. Physiol Rev 90, 607–673. 20393195 10.1152/physrev.00011.2009

[tjp12627-bib-0008] Cogswell ME , Mugavero K , Bowman BA & Frieden TR (2016). Dietary sodium and cardiovascular disease risk – Measurement matters. N Engl J Med 375, 580–586. 27248297 10.1056/NEJMsb1607161 PMC5381724

[tjp12627-bib-0009] Ecelbarger CA , Terris J , Hoyer JR , Nielsen S , Wade JB & Knepper MA (1996). Localization and regulation of the rat renal Na^+^‐K^+^‐2Cl^−^ cotransporter, BSC‐1. Am J Physiol Renal Physiol 271, F619–F628. 10.1152/ajprenal.1996.271.3.F619 8853424

[tjp12627-bib-0010] Epstein FH , Agmon Y & Brezis M (1994). Physiology of renal hypoxia. Ann N Y Acad Sci 718, 72–81; discussion 81–72. 8185253

[tjp12627-bib-0011] Féraille E & Doucet A (2001). Sodium–potassium–adenosinetriphosphatase‐dependent sodium transport in the kidney: hormonal control. Physiol Rev 81, 345–418. 11152761 10.1152/physrev.2001.81.1.345

[tjp12627-bib-0012] Fisher KA , Lee SH , Walker J , Dileto‐Fang C , Ginsberg L & Stapleton SR (2001). Regulation of proximal tubule sodium/hydrogen antiporter with chronic volume contraction. Am J Physiol Renal Physiol 280, F922–F926. 11292636 10.1152/ajprenal.2001.280.5.F922

[tjp12627-bib-0013] Frindt G , Ergonul Z & Palmer LG (2008). Surface expression of epithelial Na channel protein in rat kidney. J Gen Physiol 131, 617–627. 18504317 10.1085/jgp.200809989 PMC2391254

[tjp12627-bib-0014] Frindt G & Palmer LG (2009). Surface expression of sodium channels and transporters in rat kidney: effects of dietary sodium. Am J Physiol Renal Physiol 297, F1249–F1255. 19741015 10.1152/ajprenal.00401.2009 PMC2781327

[tjp12627-bib-0015] Frindt G & Palmer LG (2015). Acute effects of aldosterone on the epithelial Na channel in rat kidney. Am J Physiol Renal Physiol 308, F572–F578. 25520012 10.1152/ajprenal.00585.2014 PMC4360037

[tjp12627-bib-0016] Gamba G , Miyanoshita A , Lombardi M , Lytton J , Lee WS , Hediger MA & Hebert SC (1994). Molecular cloning, primary structure, and characterization of two members of the mammalian electroneutral sodium–(potassium)–chloride cotransporter family expressed in kidney. J Biol Chem 269, 17713–17722. 8021284

[tjp12627-bib-0017] Gamba G , Saltzberg SN , Lombardi M , Miyanoshita A , Lytton J , Hediger MA , Brenner BM & Hebert SC (1993). Primary structure and functional expression of a cDNA encoding the thiazide‐sensitive, electroneutral sodium‐chloride cotransporter. Proc Natl Acad Sci USA 90, 2749–2753. 8464884 10.1073/pnas.90.7.2749 PMC46173

[tjp12627-bib-0018] Gonin S , Deschênes G , Roger F , Bens M , Martin PY , Carpentier JL , Vandewalle A , Doucet A & Féraille E (2001). Cyclic AMP increases cell surface expression of functional Na,K‐ATPase units in mammalian cortical collecting duct principal cells. Mol Biol Cell 12, 255–264. 11179413 10.1091/mbc.12.2.255 PMC30941

[tjp12627-bib-0019] Graudal N , Jürgens G , Baslund B & Alderman MH (2014). Compared with usual sodium intake, low‐ and excessive‐sodium diets are associated with increased mortality: a meta‐analysis. Am J Hypertens 27, 1129–1137. 24651634 10.1093/ajh/hpu028

[tjp12627-bib-0020] Greger R ( 1985). Ion transport mechanisms in thick ascending limb of Henle's loop of mammalian nephron. Physiol Rev 65, 760–797. 2409564 10.1152/physrev.1985.65.3.760

[tjp12627-bib-0021] Greger R ( 2000). Physiology of renal sodium transport. Am J Med Sci 319, 51–62. 10653444 10.1097/00000441-200001000-00005

[tjp12627-bib-0022] Grimm PR , Lazo‐Fernandez Y , Delpire E , Wall SM , Dorsey SG , Weinman EJ , Coleman R , Wade JB & Welling PA (2015). Integrated compensatory network is activated in the absence of NCC phosphorylation. J Clin Invest 125, 2136–2150. 25893600 10.1172/JCI78558 PMC4463200

[tjp12627-bib-0023] Ha J , Daniel S , Broyles SS & Kim KH (1994). Critical phosphorylation sites for acetyl‐CoA carboxylase activity. J Biol Chem 269, 22162–22168. 7915280

[tjp12627-bib-0024] Hallows KR , Raghuram V , Kemp BE , Witters LA & Foskett JK (2000). Inhibition of cystic fibrosis transmembrane conductance regulator by novel interaction with the metabolic sensor AMP‐activated protein kinase. J Clin Invest 105, 1711–1721. 10862786 10.1172/JCI9622 PMC378514

[tjp12627-bib-0025] Holtbäck U , Aperia A & Celsi G (1993). High salt alone does not influence the kinetics of the Na^+^–H^+^ antiporter. Acta Physiol Scand 148, 55–61. 8392775 10.1111/j.1748-1716.1993.tb09531.x

[tjp12627-bib-0026] Kiil F , Aukland K & Refsum HE (1961). Renal sodium transport and oxygen consumption. Am J Physiol 201, 511–516. 13755902 10.1152/ajplegacy.1961.201.3.511

[tjp12627-bib-0027] Klemens CA , Edinger RS , Kightlinger L , Liu X & Butterworth MB (2017). Ankyrin G expression regulates apical delivery of the epithelial sodium channel (ENaC). J Biol Chem 292, 375–385. 27895120 10.1074/jbc.M116.753616 PMC5217695

[tjp12627-bib-0028] Kotchen TA , Cowley AW & Frohlich ED (2013). Salt in health and disease–a delicate balance. N Engl J Med 368, 2531–2532. 10.1056/NEJMc1305326 23802533

[tjp12627-bib-0029] Lambers Heerspink HJ , Navis G & Ritz E (2012). Salt intake in kidney disease – a missed therapeutic opportunity? Nephrol Dial Transplant 27, 3435–3442. 22942175 10.1093/ndt/gfs354

[tjp12627-bib-0030] Lassen UV & Thaysen JH (1961). Correlation between sodium transport and oxygen consumption in isolated renal tissue. Biochim Biophys Acta 47, 616–618. 13759312 10.1016/0006-3002(61)90567-4

[tjp12627-bib-0031] Layton AT , Vallon V & Edwards A (2016). Predicted consequences of diabetes and SGLT inhibition on transport and oxygen consumption along a rat nephron. Am J Physiol Renal Physiol 310, F1269–F1283. 26764207 10.1152/ajprenal.00543.2015 PMC4935777

[tjp12627-bib-0032] Leviel F , Hübner CA , Houillier P , Morla L , El Moghrabi S , Brideau G , Hassan H , Hatim H , Parker MD , Kurth I , Kougioumtzes A , Sinning A , Pech V , Riemondy KA , Miller RL , Hummler E , Shull GE , Aronson PS , Doucet A , Wall SM , Chambrey R & Eladari D (2010). The Na^+^‐dependent chloride‐bicarbonate exchanger SLC4A8 mediates an electroneutral Na^+^ reabsorption process in the renal cortical collecting ducts of mice. J Clin Invest 120, 1627–1635. 20389022 10.1172/JCI40145 PMC2860930

[tjp12627-bib-0033] Loffing J & Kaissling B (2003). Sodium and calcium transport pathways along the mammalian distal nephron: from rabbit to human. Am J Physiol Renal Physiol 284, F628–F643. 12620920 10.1152/ajprenal.00217.2002

[tjp12627-bib-0034] Loffing J , Pietri L , Aregger F , Bloch‐Faure M , Ziegler U , Meneton P , Rossier BC & Kaissling B (2000). Differential subcellular localization of ENaC subunits in mouse kidney in response to high‐ and low‐Na diets. Am J Physiol Renal Physiol 279, F252–F258. 10919843 10.1152/ajprenal.2000.279.2.F252

[tjp12627-bib-0035] Loffing J , Zecevic M , Feraille E , Kaissling B , Asher C , Rossier BC , Firestone GL , Pearce D & Verrey F (2001). Aldosterone induces rapid apical translocation of ENaC in early portion of renal collecting system: possible role of SGK. Am J Physiol Renal Physiol 280, F675–F682. 11249859 10.1152/ajprenal.2001.280.4.F675

[tjp12627-bib-0036] Lourdel S , Loffing J , Favre G , Paulais M , Nissant A , Fakitsas P , Créminon C , Féraille E , Verrey F , Teulon J , Doucet A & Deschênes G (2005). Hyperaldosteronemia and activation of the epithelial sodium channel are not required for sodium retention in puromycin‐induced nephrosis. J Am Soc Nephrol 16, 3642–3650. 16267158 10.1681/ASN.2005040363

[tjp12627-bib-0037] Mandel LJ (1986). Primary active sodium transport, oxygen consumption, and ATP: coupling and regulation. Kidney Int 29, 3–9. 3007851 10.1038/ki.1986.2

[tjp12627-bib-0038] Masilamani S , Wang X , Kim GH , Brooks H , Nielsen J , Nielsen S , Nakamura K , Stokes JB & Knepper MA (2002). Time course of renal Na‐K‐ATPase, NHE3, NKCC2, NCC, and ENaC abundance changes with dietary NaCl restriction. Am J Physiol Renal Physiol 283, F648–F657. 12217855 10.1152/ajprenal.00016.2002

[tjp12627-bib-0039] Meneton P , Jeunemaitre X , de Wardener HE & MacGregor GA (2005). Links between dietary salt intake, renal salt handling, blood pressure, and cardiovascular diseases. Physiol Rev 85, 679–715. 15788708 10.1152/physrev.00056.2003

[tjp12627-bib-0040] Morla L , Brideau G , Fila M , Crambert G , Cheval L , Houillier P , Ramakrishnan S , Imbert‐Teboul M & Doucet A (2013). Renal proteinase‐activated receptor 2, a new actor in the control of blood pressure and plasma potassium level. J Biol Chem 288, 10124–10131. 23430254 10.1074/jbc.M112.446393 PMC3617255

[tjp12627-bib-0041] O'Donnell M , Mente A , Rangarajan S , McQueen MJ , Wang X , Liu L , Yan H , Lee SF , Mony P , Devanath A , Rosengren A , Lopez‐Jaramillo P , Diaz R , Avezum A , Lanas F , Yusoff K , Iqbal R , Ilow R , Mohammadifard N , Gulec S , Yusufali AH , Kruger L , Yusuf R , Chifamba J , Kabali C , Dagenais G , Lear SA , Teo K , Yusuf S ; PURE Investigators (2014). Urinary sodium and potassium excretion, mortality, and cardiovascular events. N Engl J Med 371, 612–623. 25119607 10.1056/NEJMoa1311889

[tjp12627-bib-0042] Pfister R , Michels G , Sharp SJ , Luben R , Wareham NJ & Khaw KT (2014). Estimated urinary sodium excretion and risk of heart failure in men and women in the EPIC‐Norfolk study. Eur J Heart Fail 16, 394–402. 24464931 10.1002/ejhf.56

[tjp12627-bib-0043] Schreiber A , Shulhevich Y , Geraci S , Hesser J , Stsepankou D , Neudecker S , Koenig S , Heinrich R , Hoecklin F , Pill J , Friedemann J , Schweda F , Gretz N & Schock‐Kusch D (2012). Transcutaneous measurement of renal function in conscious mice. Am J Physiol Renal Physiol 303, F783–F788. 22696603 10.1152/ajprenal.00279.2012

[tjp12627-bib-0044] Song J , Hu X , Shi M , Knepper MA & Ecelbarger CA (2004). Effects of dietary fat, NaCl, and fructose on renal sodium and water transporter abundances and systemic blood pressure. Am J Physiol Renal Physiol 287, F1204–F1212. 15304371 10.1152/ajprenal.00063.2004

[tjp12627-bib-0045] Sorensen MV , Grossmann S , Roesinger M , Gresko N , Todkar AP , Barmettler G , Ziegler U , Odermatt A , Loffing‐Cueni D & Loffing J (2013). Rapid dephosphorylation of the renal sodium chloride cotransporter in response to oral potassium intake in mice. Kidney Int 83, 811–824. 23447069 10.1038/ki.2013.14

[tjp12627-bib-0046] Thomson SC , Deng A , Wead L , Richter K , Blantz RC & Vallon V (2006). An unexpected role for angiotensin II in the link between dietary salt and proximal reabsorption. J Clin Invest 116, 1110–1116. 16557296 10.1172/JCI26092 PMC1409739

[tjp12627-bib-0047] Tse CM , Brant SR , Walker MS , Pouyssegur J & Donowitz M (1992). Cloning and sequencing of a rabbit cDNA encoding an intestinal and kidney‐specific Na^+^/H^+^ exchanger isoform (NHE‐3). J Biol Chem 267, 9340–9346. 1374392

[tjp12627-bib-0048] Velázquez H & Wright FS (1986). Effects of diuretic drugs on Na, Cl, and K transport by rat renal distal tubule. Am J Physiol 250, F1013–F1023. 3717344 10.1152/ajprenal.1986.250.6.F1013

[tjp12627-bib-0049] Wagner CA , Loffing‐Cueni D , Yan Q , Schulz N , Fakitsas P , Carrel M , Wang T , Verrey F , Geibel JP , Giebisch G , Hebert SC & Loffing J (2008). Mouse model of type II Bartter's syndrome. II. Altered expression of renal sodium‐ and water‐transporting proteins. Am J Physiol Renal Physiol 294, F1373–F1380. 18322017 10.1152/ajprenal.00613.2007

[tjp12627-bib-0050] Walkowska A , Pawlak M , Jane SM , Kompanowska‐Jezierska E & Wilanowski T (2017). Effects of high and low sodium diet on blood pressure and heart rate in mice lacking the functional Grainyhead‐like 1 gene. Physiol Res 66, 163–165. 27782736 10.33549/physiolres.933298

[tjp12627-bib-0051] Wang YB , Leroy V , Maunsbach AB , Doucet A , Hasler U , Dizin E , Ernandez T , de Seigneux S , Martin PY & Féraille E (2014). Sodium transport is modulated by p38 kinase‐dependent cross‐talk between ENaC and Na,K‐ATPase in collecting duct principal cells. J Am Soc Nephrol 25, 250–259. 24179170 10.1681/ASN.2013040429 PMC3904566

[tjp12627-bib-0052] Welch WJ , Blau J , Xie H , Chabrashvili T & Wilcox CS (2005). Angiotensin‐induced defects in renal oxygenation: role of oxidative stress. Am J Physiol Heart Circ Physiol 288, H22–H28. 15598867 10.1152/ajpheart.00626.2004

[tjp12627-bib-0053] Yang LE , Sandberg MB , Can AD , Pihakaski‐Maunsbach K & McDonough AA (2008). Effects of dietary salt on renal Na^+^ transporter subcellular distribution, abundance, and phosphorylation status. Am J Physiol Renal Physiol 295, F1003–F1016. 18653479 10.1152/ajprenal.90235.2008 PMC2576159

